# Mechanical properties of the NiTi Memoria Leaf Spring Activated Expander (NiTi MLSAE) for maxillary transverse discrepancy correction: An *in-vitro* study

**DOI:** 10.4317/jced.56579

**Published:** 2020-02-01

**Authors:** Ronald Lowe, Steven Makowka, Kevin Manzella, Stephen Warunek, Thikriat Al-Jewair

**Affiliations:** 1 Dental Student, School of Dental Medicine, State University of New York at Buffalo, Buffalo, NY, USA; 2 Research Specialist, University at Buffalo, School of Dental Medicine, State University of New York at Buffalo, Buffalo, NY, USA; 3 Private Practice, West Seneca, NY, USA; 4 Clinical Assistant Professor, Department of Orthodontics, School of Dental Medicine, State University of New York at Buffalo, Buffalo, NY, USA; 5 Associate Professor and Graduate Program Director Department of Orthodontics, School of Dental Medicine, State University of New York at Buffalo, Buffalo, NY, USA

## Abstract

**Background:**

To determine the mechanical properties of the NiTi Memoria® Leaf Spring Activated Expander (NiTi MLSAE) in two forms, unaltered (unbent) and altered (bent) to mimic clinical use.

**Material and Methods:**

This *in-vitro* pilot study was conducted using eight NiTi MLSAE expanders (American Tooth Industries, Oxnard, California) representing four force magnitudes: 10mm 500g, 10mm 900g, 6mm 450g and 6mm 900g models. Two experiments were performed: the first tested the expanders in their unbent form and the second tested them after they were bent by one experienced technician. All expanders were adapted to a standard three dimensional printed maxillary study model. A Dillion Quantrol 500N (110lbf) load cell and a custom-made fixturing apparatus was used to determine the amount of expansive forces delivered. Prior to testing, the ligation compressing the NiTi MLSAE leaves was cut to allow the appliances to expand to their original form. Emperor™ (force) Software was used to measure the expansion forces.

**Results:**

The average expansion forces generated by the expanders were: unaltered = 897.4g (8.8N) and bent = 877.0g (8.6N) for the 10mm 900g model, unaltered = 489.5g (4.8N) and bent = 479.3g (4.7N) for the 10mm 500g model, unaltered = 458.9g (4.5N) and bent = 438.5g (4.3N) for the 6mm 450g model, and unaltered = 805.6g (7.9N) and bent = 785.2g (7.7N) for the 6mm 900g model.

**Conclusions:**

Regardless of whether the expander was straight or bent, the forces generated by the 10mm 900g, 10mm 500g and 6mm 450g Ni-Ti MLSAEs correlated with the benchmark study conducted by the manufacturer. However, the forces generated by the 6mm 900g Ni-Ti MLSAE were less than the data published by the manufacturer. Binding was observed when the expanders were manipulated to mimic clinical use, and this may account for the reported lower expansion force.

** Key words:**Maxillary expansion, NiTi, posterior crossbite, malocclusion, maxillary transverse discrepancy.

## Introduction

Maxillary Transverse Discrepancy (MTD) is a common malocclusion in primary, mixed and permanent dentitions (1). MTD can present as constriction of the inter-maxillary arch width, with or without a lingual crossbite. Lingual crossbites occur when maxillary teeth are located lingually to the mandibular teeth (2). The etiology of MTD is multifactorial, involving genetic and environmental factors (3).

Since crossbites do not self-correct, multiple management methods have been used in growing patients; among these methods, utilization of maxillary palatal expansion devices has been the most common. Few expansion devices utilizing the unique characteristics of nickel titanium (NiTi) have been manufactured. The first example of NiTi expansion was developed by Wendell Arndt in 1993 (4). Intraorally, a change from the martensitic phase to the austenitic phase produced a force of 180-300g. The second example of NiTi expansion was termed the Nitanium Palatal Expander2 (NPE2), presented by Maurice Corbett in 1997 (5). The NPE2 appliance is hygienic and compliance-free. It was reported to deliver a force of 350g at 3mm of activation (5). In 2004, the Memory Palatal Split Screw (MPSS) was introduced by Andrea Wichelhaus (6). This expander was evaluated over six months and produced dento-skeletal effects that were very similar to traditional rapid expansion screws. The MPSS has been shown to increase anterior arch width by 6.88 ± 2.47mm and posterior arch width by 7.88 ± 2.07mm. This study also demonstrated disruption of the mid-palatal suture on occlusal radiographs after being activated six times a day under a constant force of 1,224 - 1,428g (6). Patient compliance was required with recommended activations of three quarter-turns in the morning and three quarter-turns in the evening.

In 2013, Leone SpA (Florence, Italy) introduced a slow maxillary expander which utilizes a new spring-based expansion screw with a leaf-shaped active element, the NiTi Memoria® Leaf Spring Activated Expander (NiTi MLSAE). Currently, there are two force models of the NiTi MLSAE available. These include light force (500g) and medium force (900g) models with screws of 6mm or 10mm length options.

A recent retrospective study (7) assessed the clinical efficacy of the NiTi MLSAE in adolescents with a mean age of 12.72 ± 3.07 years in comparison with untreated controls from the Michigan Growth Study. The NiTi expanders were attached to the maxillary first premolars and first molars. The authors found that 1 - 1.5mm per month of expansion is expected in the molar and premolar regions. They concluded that the NiTi MLSAE is capable of obtaining adequate expansion in patients 6 - 16 years without causing significant buccal tipping when compared to untreated controls (7). Additionally, the study claims NiTi MLSAE should be considered a slow expansion device that allows for calibrated expansion on a monthly basis. Lanteri *et al.* (8) reported similar findings in younger children.

The use of NiTi spring-based expanders is very promising for orthodontics because it minimizes or eliminates the need for home activation and simplifies clinical management (8). Without the need for compliance from patients, it performs controlled expansion and avoids undesirable side effects on the permanent teeth (8).

Initially, in 2015 Leone published an article claiming: “two types of leaf springs are currently commercially available, the Light 500 gram and Medium 800 gram” (9). Shortly after the publication of this article, Leone recanted their statement and published two new forces in their 2018 catalogue – the new reported forces for the Light and Medium models were claimed to be 450g and 900g, respectively(10). This modification in forces, and whether the expanders were bent or not prior to force determination, warranted further research.

The aims for this study were to: 1) determine the reactive forces of the NiTi MLSAE by assessing compression and expansion forces under two conditions, unaltered (unbent) and altered (bent), to simulate intra-oral use; and 2) compare data from this study against the benchmark study conducted by Leone.

## Material and Methods

This is a two part *in-vitro* study that used four different force models of the NiTi MLSAE using eight expanders in total. The first part involved measuring the forces generated by the unaltered (unbent) NiTi MLSAE. The second part involved wire bending of the NiTi MLSAE to mimic intra-oral use and then measuring the forces generated by the altered (bent) appliances. All experiments were conducted by two experienced investigators (R.L., S.M.). Prior to testing, both investigators were calibrated in order to ensure validation of testing.

Part 1: Reactive forces of unaltered (unbent) NiTi MLSAE:

Unaltered NiTi MLSAE refers to a standard, straight expander without modifications for intraoral usage. Eight expanders were tested. A description of the expanders is presented in  Table 1. All expanders were obtained from the manufacturer and were optically inspected for any mechanical defects prior to testing. To determine the reactive forces, each expander was attached via clamps to the 500N (110lbf) load cell of a Dillion Quantrol TC2-i universal testing machine. Each expander model was tested 10 times for a total of 80 trial runs. One trial run consisted of fully activating the NiTi Leaf by compressing it to its maximum compression and ligating the screws with a 0.10-inch stainless steel ligature. Then the ligature was cut, letting the appliance return to its original, deactivated state.

**Table 1:**
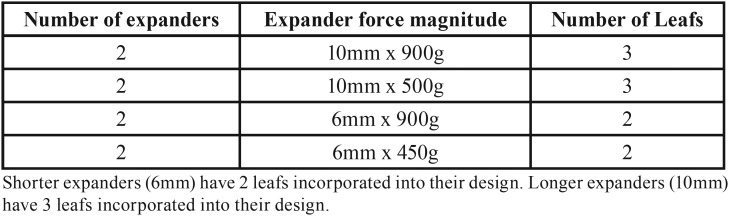
Types of NiTi MLSAE utilized.

**Figure 1:**
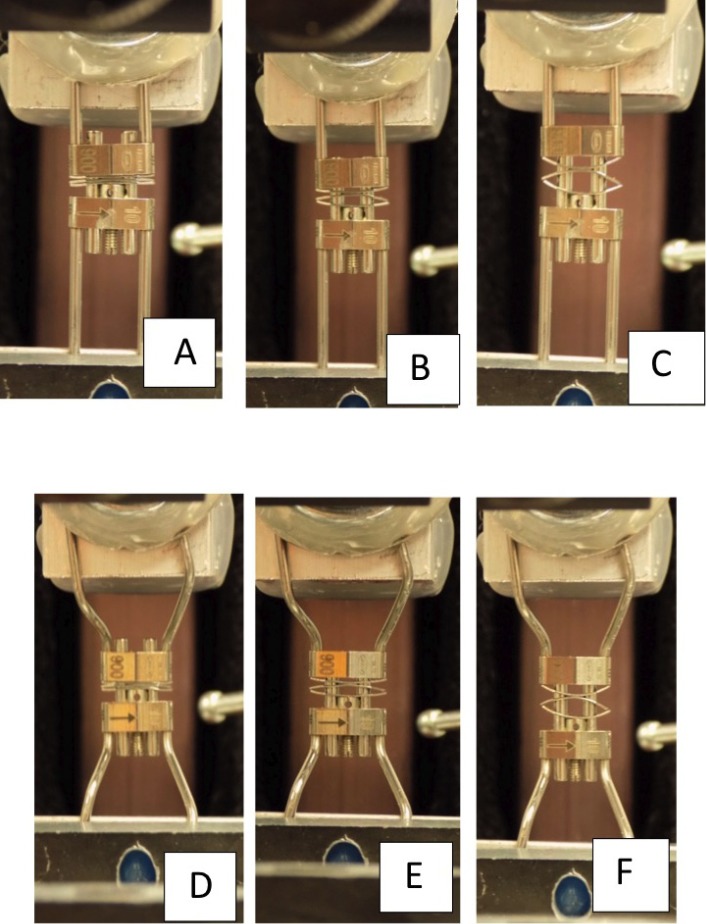
Expansion of unaltered (A-C) and altered (D-F) NiTi MLSAE.

The expansion forces were measured using Emperor™ (force) Software (DLL Version 1.13-000, Mecmesin, Sterling, VA); the control software for the Dillion Quantrol TC2-i universal testing machine. A custom force testing routine was created to determine the force displayed by allowing the NiTi MLSAE to fully expand (Fig. 1 A-C).

Part 2: Reactive forces of altered (bent) NiTi MLSAE:

The same eight expanders were used for the second experiment. The expanders were altered (bent) to mimic intra-oral use. All expanders were bent by one expert technician, at one orthodontic laboratory (Great Lakes Dental Technologies, Tonawanda, NY).

Each expander was adapted to the same three-dimensional printed resin study model. The model represented a maxillary arch that required inter-molar expansion of around 6 mm. For standardization purposes, 5 reference lines were drawn on the study model. One plane was drawn along the mid-sagittal plane, between the central incisors. Additional lines were drawn bilaterally from the mid-sagittal plane through the gingival margins of the first-premolars. 1.5mm wide grooves were placed 4mm deep in the premolars to allow the expander arms to rest at a mid-coronal level, simulating intra-oral use. Finally, a third set of lines were drawn bilaterally from the mid-sagittal plane through the gingival margin of the distolingual cusp of the first-molar. Additionally, 1.5mm wide grooves were placed 3.5mm deep in the first molars to allow room for the expander arms to rest at the mid-coronal level. After determining the position of the expander arms, a polyvinyl siloxane stop was used to standardize the position of the body of the expanders, at a height of 2.5mm from the palate. After the expanders were bent, they were re-inspected optically for any mechanical defects prior to testing.

To maintain consistency with part 1 of the study, the amount of expansive force displayed by each expander was tested with the same Dillion Quantrol TC2-i universal testing machine with a 500N (110lbf) load cell and Emperor™ Software (Fig. 1 D-F).

After recording the compression and expansion forces with the Emperor™ Software, the data were transferred to a Microsoft Excel file. The data points were then graphed for each appliance. The point of maximum expansion was determined by observing the slope of each graph. The average expansive force and standard deviation were calculated using Microsoft Excel.

## Results

**Figure 2:**
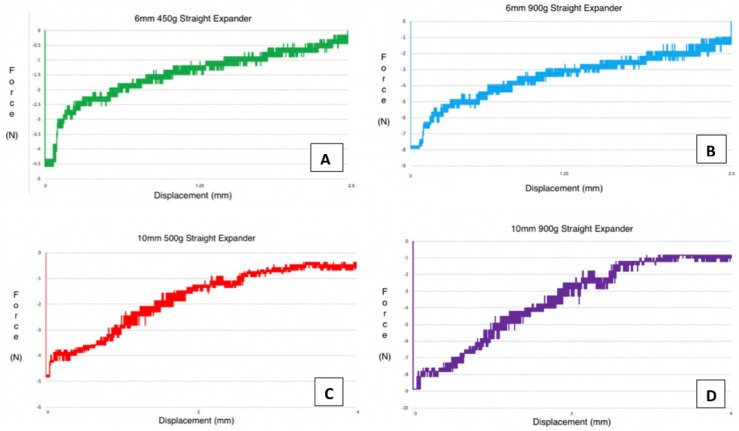
Forces generated by unaltered NiTi MLSAE.

Part 1: Unaltered NiTi MLSAE Mean Forces:

The force generated by the 6mm 450g expander was 458.9 ± 17.8g (4.5 ± 0.2N) (Fig. 2A). The force generated by the 6mm 900g expander was 805.6 ± 14.3g (7.9 ± 0.1N) (Fig. 2B). The 10mm 500g expander demonstrated a force of 489.5 ± 10.1g (4.8 ± 0.1N) (Fig.e 2C). The 10mm 900g design demonstrated a force of 897.4 ± 7.0g (8.8 ± 0.1N) (Fig. 2D).

**Figure 3:**
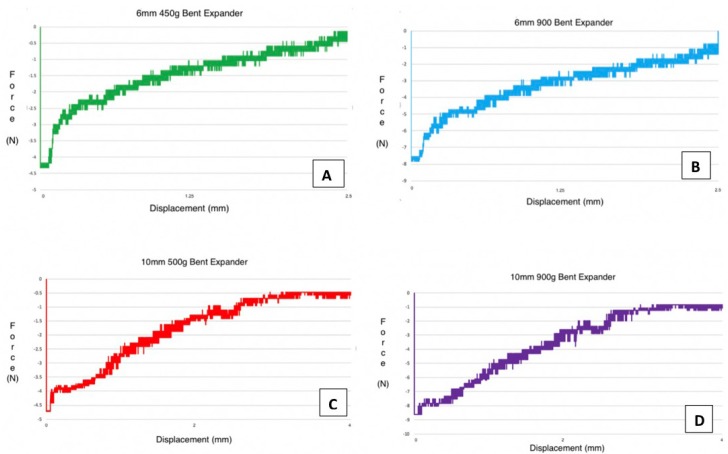
Forces generated by altered NiTi MLSAE.

Part 2: Altered NiTi MLSAE Mean Forces:

The force generated by the 6mm 450g model was 438.5 ± 12.5g (4.3 ± 0.1N) (Fig. 3A). The force measured from the 6mm 900g design was 785.2 ± 12.9g (7.7 ± 0.1N) (Fig. 3B). The force measured from the 10mm 500g model was 479.3 ± 13.8g (4.7 ± 0.1N) (Fig. 3C). The force measured from the 10mm 900g model was 877.0 ± 10.7g (8.6 ± 0.1N) (Fig. 3D).

Comparison with the benchmark study:

The benchmark study published by Leone in 2013 revealed the following information: The force generated by the 6mm “Light Force” (450g) expander was 391.3g. The force generated by the 6mm “Medium Force” (900g) expander was 826.9g. The force generated by the 10mm “Light Force” (500g) expander was 389.7g. The force generated by the 10mm “Medium Force” (900g) expander was 931.8g. A comparison of forces from this study against the benchmark study is depicted in  Table 2.

**Table 2:**
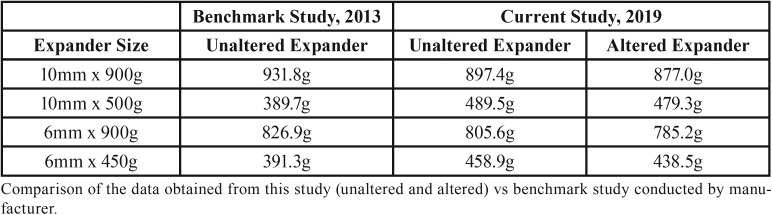
Comparison Against Benchmark.

## Discussion

This study analyzed the forces produced by four different models of the NiTi MLSAE, standardizing the same distance from the body to the palatal vault. The NiTi MLSAE is of particular interest because once the appliance is inserted, reactivation is easy and compliance is a nonissue. To our knowledge, no previous study investigated the mechanical features of the NiTi MLSAE screw and leaf spring mechanism with the body and arms. The current study demonstrated a loss of force associated with altering (bending) the appliances for intraoral use. In this study, an average loss of force of 21.35g (0.21N) was observed when the appliances were bent.

It has been reported that slow expansion is more physiologically compatible and less painful to patients than rapid expansion (11). The rate of slow expansion is similar to the rate of bone formation in the suture, leading to a less destructive process than rapid expansion. Furthermore, slow expansion is associated with less relapse and greater stability due to this more physiologic reorganization process (11).

It should be reiterated that the forces measured during this experiment are only the forces that the NiTi expander can develop. These forces are not necessarily the forces required to open the midpalatal suture. It is also important to note that the forces of the appliance intraorally will depend on the maturation stage of the midpalatal suture, bone density, inclination of anchor teeth, attachment location of expanders, skeletal age of the patient, among other factors (6,12).

The results of this study showed the 6mm 450g, 10mm 500g, and 10mm 900g designs succeeded in matching or surpassing the forces put forth by the manufacturer’s benchmark study. However, the 6mm 900g design failed to reach the reported 900g force level, reaching only 805.6g and 785.2g during the unaltered and altered tests, respectively.

When comparing the force between unaltered and altered models, an average of 21.35g (0.21N) was lost. One possible explanation for this discrepancy is binding of the appliance upon expansion. After the ligature was cut, frictional forces were observed when any significant lateral force was introduced to the expander. Although binding was observed in all models, it was most significant in the 6mm 900g model. Additionally, these expanders were designed for use in the presence of saliva. The lubrication provided from the saliva may help overcome the discrepancy in forces by reducing the observed binding.

When compared to the benchmark study published by Leone in 2013, several interesting observations can be made. First, in 2013, Leone marketed a 6mm 900g model. Today, their 6mm “medium force” model is marketed at 800g. According to the data sheets published by Leone, the average force for the 6mm 800g expander was determined to be 826.9g. This reported force is much closer to the force that was demonstrated by the 6mm Medium Force expander in our study. The 10mm medium force expander tested by Leone, also marketed at 800g in 2013, develops an average force of 931.8g. This was also very similar to the results obtained from our experiment.

In 2013, Leone marketed two “light force” models at 500g. The forces reported in their benchmark study for the 6mm 500g expander was 391.3g and the reported force of the 10mm 500g expander was 389.7g. Both of these forces were less than the forces obtained from our study.

Our study illustrates that there may be a discrepancy in the actual force delivered from each expander and the values marketed by the manufacturer. One reason for the discrepancy noted from our current study and the manufacturer’s benchmark study may be attributed to the design of the testing apparatus. In our study, the testing apparatus was carefully designed to ensure the expanders were directly in line with the vertical load cell of the universal testing machine. Without seeing the testing apparatus that the manufacturer used in 2015, it is impossible to know if the loading was not uniaxial.

When a clinician is choosing an expander, it is important to note that the value marketed by the manufacturer is an estimate rather than a definite value – a more appropriate marketing campaign would advertise these as “light” or “medium” force expanders with a range of 400-500g and 800-900g respectively. Regardless of the marketed value, the larger expander with the most force (10mm, medium force version) is recommended for more skeletally mature patients.

Study limitations

The testing environment for the expanders was the most significant limiting factor of this *in-vitro* study, which attempted to predict the devices’ behavior under clinical conditions. Although the testing conditions were the same as those put forth by the manufacturer (23˚C), a more reliable testing environment would have included a warm-water bath to mimic the intraoral environment (35˚C). Testing these appliances clinically, or even submerged in water, would allow us to better predict the effects of saliva on the appliances. For instance, saliva may lubricate the expanders and help overcome the binding that was observed. Additionally, the small sample size (8 expanders) used for this *in-vitro* study was another limitation. In order to get a more accurate representation of the force generated by these expanders, a larger sample sized should be used.

## Conclusions

1. Bent NiTi MLSA expanders generate less forces (13.2-20.4g) than unbent ones.

2. Regardless of whether the expander was unaltered or bent, the forces generated by the 10mm 900g, 10mm 500g, and 6mm 450g Ni-Ti MLSAEs correlated with the forces marketed by the manufacturer.

3. The forces generated by the 6mm 900g Ni-Ti MLSAE were less than the data published by the manufacturer.

4. Further research is warranted to assess the effects of saliva on binding of the expanders.
